# Quality of life and depression 3 months after intracerebral hemorrhage

**DOI:** 10.1002/brb3.1270

**Published:** 2019-03-24

**Authors:** Hanne Sallinen, Tiina Sairanen, Daniel Strbian

**Affiliations:** ^1^ Department of Neurology Helsinki University Hospital and University of Helsinki Helsinki Finland

**Keywords:** depression, intracerebral hemorrhage, quality of life

## Abstract

**Objectives:**

Quality of life (QoL) after intracerebral hemorrhage (ICH) is poorly known. This study investigated factors affecting QoL and depression after spontaneous ICH.

**Materials and Methods:**

This prospective study included patients admitted to Helsinki University Hospital between May 2014 and December 2016. Health‐related QoL (HRQoL) at 3 months after ICH was measured using the European Quality of Life Scale (EQ‐5D‐5L), and the 15D scale. Logistic regression analyses were used to test factors affecting HRQoL. EQ‐5D‐5L anxiety/depression dimension was used to analyze factors associated with anxiety/depression.

**Results:**

Of 277 patients, 220 were alive, and sent QoL questionnaire. The questionnaire was returned by 124 patients. Nonreturners had more severe strokes with admission National Institutes of Health Stroke Scale (NIHSS) 7.8 (IQR 3.0–14.8) versus 5.0 (IQR 2.3–11.0); *p* = 0.018, and worse outcome assessed as modified Rankin Scale 3–5 at 3 months 59.4% versus 44.4% (*p* = 0.030). Predictors for lower HRQoL by both scales were higher NIHSS with OR 1.28 (95% CI 1.13–1.46) for EQ‐5D‐5L, and OR 1.28 (1.15–1.44) for 15D, older age (OR 1.10 [1.03–1.16], and OR 1.09 [1.03–1.15]), and chronic heart failure (OR 18.12 [1.73–189.27], and OR 12.84 [1.31–126.32]), respectively. Feeling sad/depressed for more than 2 weeks during the year prior to ICH was predictor for lower EQ‐5D‐5L (OR 10.64 [2.39–47.28]), and history of ICH for lower 15D utility indexes (OR 11.85 [1.01–138.90]). Prior feelings of sadness/depression were associated with depression/anxiety at 3 months after ICH with OR 3.62 (1.14–11.45).

**Conclusions:**

In this cohort of ICH patients with milder deficits, HRQoL was affected by stroke severity, comorbidities and age. Feelings of depression before ICH had stronger influence on reporting depression/anxiety after ICH than stroke severity‐related and outcome parameters. Thus, simple questions on patient's premorbid feelings of sadness/depression could be used to identify patients at risk of depression after ICH for focusing follow‐up and treatment.

## 1 INTRODUCTION

Intracerebral hemorrhage (ICH) is the second most common type of stroke (van Asch et al., 2010). As many as 40.4% of the patients die within the first month, and many of the survivors are left with disabilities (van Asch et al., 2010), having a major effect on the life of the patients and caregivers. Stroke increases risk of depression; depression is also a risk factor for stroke (Dong, 2012). ICH patients have been shown to have less favorable outcome compared to ischemic stroke, and they are left with more severe deficits (Sennfält, Norrving, Petersson, & Ullberg, 2018). In addition to risk for recurrent ICH, survivors are at risk for thrombotic events, such as ischemic stroke, raising concerns for the benefits and risks of secondary prevention, such as antiplatelet therapy (Poon, Fonville, & Salman, 2014; Wani, Nga, & Navaratnasingham, 2005). After ischemic stroke, there are many modifiable factors to prevent recurrence (Diener, Ntaios, O'Donnell, & Easton, 2018; Laloux, 2014). The most important etiological factor to control after ICH is hypertension; however, no ICH specific secondary preventive medications exist to date (Veltkamp & Purrucker, 2017). Seizures are more common after ICH than ischemic stroke (Christensen, Mayer, Ferran, & Kissela, 2009; Faught, Peters, Bartolucci, Moore, & Miller, 1989; Lahti et al., 2017). Such stroke sequelae and lack of effective secondary prevention can feel burdensome to the patient and caregivers, and affect their quality of life.

There are several studies on health‐related quality of life (HRQoL) after stroke (Chang et al., 2016; Delcourt et al., 2010; Hackett, Duncan, Anderson, Broad, & Bonita, 2000), but few focus on ICH patients. Age, length of hospital stay, and motor function at discharge have been shown to predict HRQoL after stroke at 6 months (Chang et al., 2016). Another study reported baseline National Institutes of Health Stroke Scale (NIHSS) ≥14 versus <14 to have the strongest association with poor HRQoL 3 months after ICH. Also older age, use of antithrombotics, larger and deep ICH, intraventricular hemorrhage (IVH), early worsening of the neurological deficit, and completion of the questionnaire by proxy responder associated with worse HRQoL (Christensen et al., 2009; Faught et al., 1989; Lahti et al., 2017). Hence, these parameters are associated mostly with the severity of the index ICH.

Post‐ICH depression has been shown to have a negative impact on outcome unrelated to initial hemorrhage severity (Stern‐Nezer et al., 2017). EQ‐5D‐5L (Herdman et al., 2011) and 15D questionnaires are developed to measure HRQoL, and they have been validated, and used in cohorts with stroke patients (Golicki et al., 2015; Hunger, Sabariego, Stollenwerk, Cieza, & Leidl, 2012; Naess, Lunde, & Brogger, 2012; Puumalainen, Numminen, Elonheimo, Roine, & Sintonen, 2016; Sintonen, 2001). EQ‐5D‐5L and 15D define health in five (EQ‐5D‐5L), and 15 (15D) dimensions, including motor as well as mental functioning. In earlier studies, many of the factors that associate with worse HRQoL are also predictors of worse functional outcome. Other components of quality of life, such as mood and anxiety, remain largely uninvestigated.

In this study, we assessed the quality of life and depression after ICH in a prospective cohort, and addressed such factors affecting the subjective quality of life that are not included in traditional outcome measures.

## 2 MATERIALS AND METHODS

This study is part of a prospective study on genetic and environmental risk factors of ICH. We aimed to recruit all consecutive patients admitted to Helsinki University Hospital, Finland, between May 2014 and December 2016 with findings of primarily appearing ICH on admission imaging. We subsequently excluded hemorrhages related to tumor, trauma, ischemic stroke, vascular malformations, and other structural abnormalities.

To participate, the patient or his/her proxy had to give an informed signed consent. If the patient was unable to write, the consent could be given orally in the presence of a witness unrelated to the study. We used structured questionnaires on profession, feelings of depression, smoking, and alcohol consumption before ICH. The questionnaire was given upon recruitment in hospital, that is, in the acute/subacute phase, and if not returned, they were sent to the patient's home address. The return time of questionnaires were not registered systematically. Medical history was obtained by using the combination of questionnaires and medical records. Admission parameters such as blood pressure, NIHSS, and Glascow Coma Scale (GCS) were obtained from the electronic medical charts. Hematoma size was measured from first head CT after arrival, and the size was calculated using ABC/2 method, where A, B, and C are the largest perpendicular measures of the hematoma on CT images.

Living and working status, modified Rankin Scale (mRS), and Barthel Index (BI) were evaluated at 3 months (±1 week) after ICH by a combination of revisiting the electronic medical charts and telephone interview (HS). The patients were interviewed via telephone, or alternatively, if the corresponding information on the outcome parameters were recorded in their electronic medical charts, these were collected. If the patient was reached later, he/she was asked to recall the situation at 3 months. As the medical files in Finland are very comprehensive (including physiotherapists', occupational therapists', and neuropsychologists' evaluations), the information is found to a vast extent in the patients' files. At the same time point, the patients were sent 15D and EQ‐5D‐5L questionnaires. EQ‐5D‐5L is a validated measure developed by EuroQol Group (www.euroqol.org), defining health in terms of five dimensions: Mobility, Self‐ Care, Usual Activities, Pain/Discomfort, and Anxiety/Depression, each of them having five response levels (no problems, slight, moderate, severe, and extreme problems). The EQ‐5D‐5L utility score is an index, which combines the five dimensions into one score, which is calculated using population‐based preference weights. One represents perfect health, 0 death, and negative values health states considered worse than death. As there was no population‐based utility index dataset for EQ‐5D‐5L for the Finnish population, the utility index was calculated using a crosswalk dataset from the EQ‐5D‐3L, provided by the EuroQol Research Foundation. 15D is a 15‐dimension (Mobility, Vision, Hearing, Breathing, Sleeping, Eating, Speech, Excretion, Usual activities, Mental function, Discomfort and symptoms, Depression, Distress, Vitality, Sex) health state descriptive questionnaire with five levels for each question, 0 being the worst, and five the best possible. 15D yields a single index (15D score) on 0–1 scale, representing the overall HRQoL. The index is calculated using a set of population‐based utility weights.

The patients were divided into two utility index groups using the median utility indexes (low or high). For EQ‐5D‐5L, the HRQoL was considered low, if the index was ≤0.590 (median), and high, if the index was >0.590, and for 15D ≤0.817 and >0.817, accordingly. For the EQ‐5D‐5L depression/anxiety question, the patients were divided into two groups: reporting symptoms of depression/anxiety or not. We compared the baseline and outcome characteristics between each two groups using univariate regression analysis. Variables with *p* < 0.2 in the univariate regression model were chosen for the multivariable binary regression model for HRQoL, and variables with *p* < 0.1 for depression/anxiety. For the multivariable models on EQ‐5D‐5L and 15D utility indexes, we tested associations of baseline characteristics with the quality of life, and thus the outcome parameters were not included in the analyses. We also compared the characteristics of the patient groups with and without (missing) data on quality of life to test for selection bias.

Statistical analyses were made using SPSS v.24 (IBM, Armonk, NY); the multivariable analysis for depression/anxiety was made using R Package “brglm” to be able to handle the situation with no cases in one group; for QoL index values we used logistic regression analysis (Forward LR). We express categorical variables as counts (%), continuous variables with normal distribution as mean (SE), and continuous variables not normally distributed as median (IQR) values. The differences between the groups were calculated using the χ2 test, the *t* test, or the Mann–Whitney *U* test as appropriate.

This study has been approved by the institutional review board and local ethical committee of Helsinki University Hospital (11.12.2013 311/13103/01/2013 and 12.10.2016 HUS/1662/2016).

## 3 RESULTS

Of 456 ICH patients treated in our hospital during the study period, 291 (63.8%) consented to participate in the study, and 277 of those did not have any exclusive secondary reason for ICH during the follow‐up. The median age was 70.5 years (IQR 62.0–78.0 years), median NIHSS 5.0 (IQR 2.3–11.0), median GCS 15 (IQR 14.0–15.0), and the median ICH volume 8.9 ml (IQR 2.6–19.0 ml). The mortality at 3 months was 20.2% (56 patients), and one foreign patient was lost to follow‐up. One hundred and twenty‐four out of 220 survived patients (56.4%) returned the questionnaire.

Supplemental Table S1 shows baseline comparison between patients with and without (non‐responders) data on HRQoL. Patients not completing the questionnaire had more severe strokes (higher NIHSS; median 7.8; IQR 3.0–14.8 vs. median 5.0; IQR 2.3–11.0; *p* = 0.018), and worse outcome at 3 months: larger proportion of patients with mRS 3–5 (59.4% vs. 44.4%; *p* = 0.030), lower BI (median 95.0; IQR 35.0–100.0 vs. median 100.0; IQR 85.0–100.0; *p* = 0.013), and were less likely to live at home (54.7% vs. 77.4%; *p* < 0.001). Other parameters showed no significant differences.

In the general Finnish population, the EQ‐5D index values range from 0.909 (age group 25–34) to 0.583 (age group 75+), the average value being 0.800 (Self‐Reported Population Health: An International Perspective based on EQ‐5D, 2014). Median EQ‐5D‐5L utility index was 0.590 in the present ICH cohort. For 7 (5.6%) patients the index could not be calculated due to incomplete answers. Median 15D utility index was 0.817. For 15D scores there were 16 (12.9%) patients with incomplete data. Supplemental Figure S1 shows the distribution of the EQ‐5D‐5L and 15D utility index values.

Table 1 shows comparison of the patients with low (≤0.590) and high (>0.590) EQ‐5D‐5L utility indexes. Lower HRQoL associated with consent given by patient's proxy, history of COPD or asthma, frequently felt home‐related stress, lower education level, higher NIHSS and lower GCS scores on admission, higher baseline ICH volumes, deep ICH location, and DNR orders during hospital stay. Patients with lower HRQoL had lower BI, higher mRS, and were less likely to live at home at 3 months and 1 year. Patients with better HRQoL had more likely had cancer, including curatively treated forms.

**Table 1:** Comparison of the patients with low and high EQ‐5D‐5L utility index (*n* = 117)^a^

Variable	Low (≤0.590) *n* = 59	High (>0.590) *n* = 58	*p* value
Age, y	70.0 (62.0–80.0)	70.5 (61.8–75.0)	0.195
Male	29 (49.2)	32 (55.2)	0.515
Agreement by proxy	25 (42.4)	8 (13.8)	**0.001**
mRS 3–5 prior to ICH	4 (6.8)	0 (0.0)	0.119
Diabetes	9 (15.3)	7 (12.1)	0.616
History of ischemic stroke	9 (15.3)	6 (10.3)	0.427
History of any ICH	5 (8.5)	2 (3.4)	0.439
COPD/asthma	9 (15.3)	2 (3.4)	**0.029**
Dementia	3 (5.1)	1 (1.7)	0.619
Coronary artery disease	9 (15.3)	3 (5.2)	0.072
Atrial fibrillation	15 (25.4)	14 (24.1)	0.872
Chronic heart failure	7 (11.9)	1 (1.7)	0.061
History of cancer	9 (15.3)	18 (31.0)	**0.043**
Antiplatelet treatment	13 (22.0)	10 (17.2)	0.514
Anticoagulant treatment	13 (22.0)	12 (20.7)	0.859
Antihypertensive medication	32 (54.2)	29 (50.0)	0.646
Antidepressant medication	3 (5.1)	0 (0.0)	0.244
Sad or depressed for more than 2 weeks during the year prior to ICH^b^	14 (30.4)	6 (11.1)	0.160
Any alcohol use within 1 year prior to ICH^b^	49 (86.0)	45 (78.9)	0.325
No or little stress at home^b^	39 (72.2)	47 (90.4)	**0.017**
Living alone	24 (40.7)	14 (24.1)	0.056
Married or registered relationship^b^	32 (56.1)	36 (63.2)	0.445
Occupational status, working	10 (16.9)	14 (24.1)	0.336
High education (Master or Bachelor's degree)^b^	10 (17.9)	20 (35.1)	**0.038**
NIHSS on admission	10.0 (3.0–15.0)	4.0 (2.0–6.0)	**<0.001**
GCS on admission	15.0 (13.0–15.0)	15.0 (14.0–15.0)	**<0.001**
Baseline ICH volume, mL	10.9 (4.5–23.0)	7.2 (1.9–16.2)	**0.037**
ICH location			**0.001**
ICH location, lobar	8 (13.6)	24 (41.4)	
ICH location, deep supratentorial	48 (81.4)	30 (51.7)	
ICH location, infratentorial	3 (5.1)	4 (6.9)	
Deterioration (increase in NIHSS at least 4 points) within 1 week of ICH	5 (8.5)	1 (1.7)	0.207
Acute myocardial infarction or heart failure within 1 week of ICH	2 (3.4)	0 (0.0)	0.496
Any neurosurgery	4 (6.8)	1 (1.7)	0.364
DNR orders in hospital	12 (20.3)	3 (5.2)	**0.014**
mRS at 3 months			**<0.001**
mRS 0	0 (0.0)	5 (8.6)	
mRS 1	2 (3.4)	11 (19.0)	
mRS 2	16 (27.1)	34 (58.6)	
mRS 3	15 (25.4)	5 (8.6)	
mRS 4	16 (27.1)	3 (5.2)	
mRS 5	10 (16.9)	0 (0.0)	
Barthel index at 3 months	90.0 (55.0–100.0)	100.0 (100.0–100.0)	**<0.001**
Living at home at 3 months	36 (61.0)	55 (94.8)	**<0.001**
Working at 3 months	2 (3.4)	2 (3.4)	1.000

Note

Data are *n* (%), mean (SE), or median (IQR).

mRS: modified Rankin Scale; ICH: intracerebral hemorrhage; COPD: chronic obstructive pulmonary disease; NIHSS: the National Institutes of Health Stroke Scale; GCS: Glasgow Coma Scale; DNR: do not resuscitate.

a n = 7 exclusion due to incomplete answers.

b Missing data for sad or depressed for more than 2 weeks in 17 patients (14.5%), alcohol use in three patients (2.6%), no or little stress at home in 11 patients (9.4%), marital status in three patients (2.6%), high education in four patients (3.4%).

The bold values indicate *P* < 0.05.

There were some intergroup differences associated with the HRQoL based on 15D score (Table 2). The patients with lower scores were older, were more often on antihypertensive medication, less likely lived at home at 3 months and 1 year, and their consent was more often signed by patient's proxy. They also had lower education level, higher NIHSS, lower GCS, larger baseline ICH volumes, more often DNR orders in hospital, higher mRS, and lower BI at 3 months. Supplemental Figure S2 shows the EQ‐5D‐5L and 15D utility indexes in different mRS groups.

**Table 2:** Comparison of the patients with low and high 15D score (*n* = 108)^a^

Variable	Low (≤0.817) *n* = 54	High (>0.817) *n* = 54	*p* value
Age, y	72.0 (62.8–80.0)	68.0 (60.5–75.0)	**0.035**
Male	29 (53.7)	31 (57.4)	0.699
Agreement by proxy	27 (50.0)	6 (11.1)	**<0.001**
mRS 3–5 prior to ICH	2 (3.7)	0 (0.0)	0.495
Diabetes	9 (16.7)	7 (13.0)	0.588
History of ischemic stroke	8 (14.8)	7 (13.0)	0.781
History of any ICH	6 (11.1)	1 (1.9)	0.113
COPD or asthma	6 (11.1)	2 (3.7)	0.270
Dementia	3 (5.6)	0 (0.0)	0.243
Coronary artery disease	6 (11.1)	3 (5.6)	0.489
Atrial fibrillation	16 (29.6)	11 (20.4)	0.267
Chronic heart failure	6 (11.1)	1 (1.9)	0.113
History of cancer	10 (18.5)	14 (25.9)	0.355
Antiplatelet treatment	11 (20.4)	10 (18.5)	0.808
Anticoagulant treatment	14 (25.9)	9 (16.7)	0.240
Antihypertensive medication	34 (63.0)	23 (42.6)	**0.034**
Antidepressant medication	3 (5.6)	0 (0.0)	0.243
Sad or depressed for more than 2 weeks during the year prior to ICH^b^	8 (19.0)	10 (19.6)	0.946
Any alcohol use (within 1 year prior ICH)^b^	44 (84.6)	41 (77.4)	0.344
No or little stress at home^b^	37 (75.5)	43 (84.3)	0.271
Living alone	20 (37.0)	11 (20.4)	0.056
Married or registered relationship^b^	31 (59.6)	38 (70.4)	0.246
Occupational status, working	9 (16.7)	15 (27.8)	0.165
High education (Master or Bachelor's degree)^b^	9 (17.6)	22 (41.5)	**0.008**
NIHSS on admission	9.0 (3.8–15.5)	3.0 (2.0–5.0)	**<0.001**
GCS on admission	14.5 (13.0–15.0)	15.0 (15.0–15.0)	**<0.001**
Baseline ICH volume, mL	13.6 (6.2–24.5)	4.5 (1.7–16.0)	**0.002**
ICH location			0.202
ICH location, lobar	11 (20.4)	20 (37.0)	
ICH location, deep supratentorial	39 (72.2)	31 (57.4)	
ICH location, infratentorial	4 (7.4)	3 (5.6)	
Any neurosurgery	5 (9.3)	0 (0.0)	0.057
DNR orders in hospital	14 (25.9)	2 (3.7)	**0.001**
Deterioration (increase in NIHSS at least 4 points) within 1 week of ICH	5 (9.3)	1 (1.9)	0.205
Acute myocardial infarction or heart failure within 1 week of ICH	2 (3.7)	0 (0.0)	0.495
mRS at 3 months			**<0.001**
mRS 0	0 (0.0)	4 (7.4)	
mRS 1	2 (3.7)	11 (20.4)	
mRS 2	14 (25.9)	33 (61.1)	
mRS 3	15 (27.8)	4 (7.4)	
mRS 4	13 (24.1)	2 (3.7)	
mRS 5	10 (18.5)	0 (0.0)	
Barthel Index at 3 months	92.5 (53.8–100.0)	100 (100.0–100.0)	**<0.001**
Living at home at 3 months	33 (61.1)	52 (96.3)	**<0.001**
Working at 3 months	1 (1.9)	3 (5.6)	0.618

Note

Data are *n* (%), mean (SE), or median (IQR).

mRS: modified Rankin Scale; ICH: intracerebral hemorrhage; COPD: chronic obstructive pulmonary disease; NIHSS: the National Institutes of Health Stroke Scale; GCS: Glasgow Coma Scale; DNR: do not resuscitate.

a n = 16 exclusion due to incomplete answers.

b Missing data for BMI in four patients (3.7%), sad or depressed for more than 2 weeks in 15 patients (13.9%), alcohol use in three patients (2.8%), no or little stress at home in eight patients (7.4%), marital status in three patients (1.9%), high education in four patients (3.7%).

The bold values indicate *P* < 0.05.

In multivariable regression analyses, chronic heart failure, higher NIHSS on arrival, and older age were predictors for lower HRQoL in both EQ‐5D‐5L and 15D. Additionally, feeling sad or depressed for more than 2 weeks during the year prior to ICH was an independent predictor for lower EQ‐5D‐5L utility index, and a history of any ICH for lower 15D score (Table 3). For EQ‐5D‐5L binary logistic regression analysis, Nagelkerke R Square was 0.493, indicating, that the model included approximately 50% of factors influencing EQ‐5D‐5L utility index value, and Hosmer and Lemeshow Goodness of Fit was 0.814. For 15D binary logistic regression analysis Nagelkerke R Square was 0.473, and Hosmer and Lemeshow Goodness of Fit was 0.951.

**Table 3:** Multivariable binary logistic regression analysis for predictors of decreased a) EQ‐5D‐5L utility index, and b) 15D score at 3 months^a^

Variable	OR (95% CI)	*p* value
Predictor for decreased EQ‐5D‐5L utility index^b^		
Chronic heart failure	18.12 (1.73–189.27)	0.016
History of cancer	0.20 (0.053–0.77)	0.019
NIHSS at arrival	1.28 (1.13–1.46)	<0.001
Age per year	1.10 (1.03–1.16)	0.002
Sad or depressed for more than 2 weeks during the year prior ICH	10.64 (2.39–47.28)	0.002
Predictor for decreased 15D utility index^c^		
Chronic heart failure	12.84 (1.31–126.32)	0.029
NIHSS at arrival	1.28 (1.15–1.44)	<0.001
Age per year	1.09 (1.03–1.15)	0.003
History of any ICH	11.85 (1.01–138.90)	0.049

Note

NIHSS: the National Institutes of Health Stroke Scale; ICH: intracerebral hemorrhage.

a 95 patients included in analysis due to missing data (EQ‐5D‐5L); 104 patients included in analysis due to missing data (15D).

b Adjusted for: agreement by proxy, mRS 3–5 prior to ICH, COPD/asthma, coronary artery disease, no or little stress at home, living alone, high education, GCS on admission, baseline ICH volume, ICH location, DNR orders in hospital.

c Adjusted for: agreement by proxy, antihypertensive medication, living alone, occupational status, high education, GCS on admission, baseline ICH volume, any neurosurgery, DNR orders in hospital.

As seen in Table 4, feelings of depression/anxiety at 3 months after ICH were more common among patients with mRS 3–5 at 3 months after ICH, patients with dementia before ICH, patients with DNR orders during hospital stay, and patients having been feeling sad or depressed during the year prior to ICH. However, in multivariable analysis, the only independent predictor for feelings of depression and/or anxiety at 3 months after ICH was patient reported depression in the year prior to ICH (Table 5). Only four (3.7%) patients had been treated with antidepressants, and 1 (0.9%) patient with psychotherapy.

**Table 4:** Comparison of the patients with reported depression and/or anxiety in EQ‐5D‐5L 3 months after ICH (*n* = 122)^a^

Variable	Not depressed *n* = 68	Depressed *n* = 54	*p* value
Age, y	69.5 (62.3–75.8)	72.5 (59.8–79.3)	0.335
Male	34 (50.0)	30 (55.6)	0.542
Agreement by proxy	18 (26.5)	18 (33.3)	0.409
mRS 3–5 prior to ICH	1 (1.5)	3 (5.6)	0.321
Diabetes	10 (14.7)	6 (11.1)	0.559
History of ischemic stroke	8 (11.8)	7 (13.0)	0.841
History of any ICH	2 (2.9)	5 (9.3)	0.239
COPD or asthma	4 (5.9)	7 (13.0)	0.212
Dementia	0 (0.0)	5 (9.3)	**0.015**
Coronary artery disease	4 (5.9)	8 (14.8)	0.100
History of atrial fibrillation	15 (22.1)	16 (29.6)	0.340
Chronic heart failure	3 (4.4)	5 (9.3)	0.464
History of cancer	15 (22.1)	13 (24.1)	0.793
Antiplatelet treatment	15 (22.1)	10 (18.5)	0.630
Anticoagulant treatment	11 (16.2)	15 (27.8)	0.120
Antihypertensive medication	35 (51.5)	29 (53.7)	0.806
Antidepressant medication	2 (2.9)	1 (1.9)	1.000
Sad or depressed for more than 2 weeks during the year prior to ICH^b^	6 (10.0)	15 (33.3)	**0.003**
Alcohol use (within 1 year prior to ICH)^b^	55 (83.3)	43 (81.1)	0.754
No or little stress at home^b^	53 (86.9)	37 (74.0)	0.085
Living alone	23 (33.8)	16 (29.6)	0.622
Married or registered relationship^b^	40 (59.7)	33 (63.5)	0.676
Occupational status, working	13 (19.1)	13 (24.1)	0.507
High education (Master or Bahelor's degree)^b^	21 (31.8)	10 (19.2)	0.123
NIHSS on admission	5 (2.3–9.8)	6 (2.0–13.3)	0.262
GCS on admission	15 (14.0–15.0)	15 (13.8–15.0)	0.683
Baseline ICH volume, mL	8.0 (1.9–16.4)	12.0 (4.0–21.9)	0.082
ICH location			0.555
ICH location, lobar	22 (32.4)	13 (24.1)	
ICH location, deep supratentorial	42 (61.8)	38 (70.4)	
ICH location, infratentorial	4 (5.9)	3 (5.6)	
ICH evacuation/any neurosurgery	1 (1.5)	4 (7.4)	0.169
DNR orders in hospital	4 (5.9)	12 (22.2)	**0.008**
Deterioration (increase in NIHSS at least 4 points) within 1 week of ICH	5 (7.4)	2 (3.7)	0.462
Acute myocardial infarction or heart failure within 1 week of ICH	0 (0.0)	2 (3.7)	0.194
mRS at 3 months			0.175
mRS 0	4 (5.9)	1 (1.9)	
mRS 1	10 (14.7)	3 (5.6)	
mRS 2	31 (45.6)	20 (37.0)	
mRS 3	10 (14.7)	12 (22.2)	
mRS 4	8 (11.8)	13 (24.1)	
mRS 5	5 (7.4)	5 (9.3)	
mRS 3–5 at 3 months	23 (33.8)	30 (55.6)	**0.016**
Barthel Index at 3 months	100 (91.3–100.0)	97.5 (80.0–100.0)	0.164
EQ−5D−5L utility index at 3 months^b^	0.711 (0.577–1.000)	0.517 (0.341–0.588)	**<0.001**
15D score at 3 months^b^	0.885 (0.769–0.941)	0.770 (0.588–0.827)	**<0.001**
Living at home at 3 months	54 (79.4)	42 (77.8)	0.827
Working at 3 months	2 (2.9)	2 (3.7)	1.000

Note

Data are *n* (%), mean (SE), or median (IQR).

mRS: modified Rankin Scale; ICH: intracerebral hemorrhage; COPD: chronic obstructive pulmonary disease; NIHSS: the National Institutes of Health Stroke Scale; GCS: Glasgow Coma Scale; DNR: do not resuscitate.

a n = 2 exclusion due to incomplete answers.

b Missing data for BMI in five patients (4.1%), sad or depressed for more than 2 weeks in 17 patients (13.9%), alcohol use in three patients (2.5%), no or little stress at home in 11 patients (9.0%), marital status in three patients (2.5%), high education in four patients (3.3%), EQ‐5D index in five patients (4.1%), 15D index in 14 patients (11.5%).

The bold values indicate *P* < 0.05.

**Table 5:** Multivariable binary logistic regression analysis for associations with depression/anxiety in EQ‐5D‐5L at 3 months (*n* = 122)^a^

Variable	OR (95% CI)	*p* value
Sad or depressed for more than 2 weeks during the year prior ICH	3.62 (1.14–11.45)	**0.029**
Dementia prior to ICH	16.65 (0.50–549.45)	0.11
DNR orders in hospital	4.87 (0.99–23.95)	0.051
Stress at home prior to ICH	3.01 (0.86–10.58)	0.086
Baseline ICH volume	0.99 (0.96–1.03)	0.53
mRS 3–5 at 3 months	1.90 (0.75–4.80)	0.17

Note

DNR: do not resuscitate; ICH: intracerebral hemorrhage.

a 102 patients included in analysis due to missing data.

The bold value indicates *P* < 0.05.

## 4 DISCUSSION

As previously shown (Christensen, Mayer, & Ferran, 2009; Delcourt et al., 2017), and also verified in our study, baseline stroke severity measured by NIHSS and ICH volume have a strong impact on the HRQoL after ICH. Of the comorbidities, asthma/COPD and chronic heart failure were associated with worse HRQoL, as earlier studies also imply (Chung & Han, 2017; Iqbal, Francis, Reid, Murray, & Denvir, 2010). As the consent given by proxy—the patient not being able to consent him/herself—indicates more severe stroke, it is not surprising that it associates with worse HRQoL. As also seen in general population, QoL has an inverse association with older age (Self‐Reported Population Health: An International Perspective based on EQ‐5D, 2014). Measured by EQ‐5D‐5L, patients with history of cancer reported better HRQoL. This is likely to be biased due to including and not separating also benign and curatively treated cancers.

Feelings of depression during the year before ICH were shown to be associated with mood problems also after ICH. This is in line with a study on subarachnoid hemorrhage (SAH) patients, where pre‐SAH depression and premorbid psychosocial stress were associated with depression at 3 months (Kreiter et al., 2013). Depression is known to be common among dementia patients (Meyers, 1998), and in our study, all patients with dementia reported feelings of depression and/or anxiety after ICH. However, few patients had dementia, and dementia had no statistically significant association with depression/anxiety after ICH. Other comorbidities did not have any significant effect on depression after ICH. Antidepressant medication prior to ICH was not common in either group, and few patients reporting depression before ICH had antidepression treatment during the past year. Living with family or marital status was not associated with mood problems. As expected, patients with worse outcome on mRS reported more depression and/or anxiety after ICH. However, feeling sad or depressed before ICH was the only independent predictor for depression and/or anxiety after ICH.

Since depression after ICH has been shown to have a negative impact on outcome unrelated to initial hemorrhage severity (Stern‐Nezer et al., 2017), it would be important to find the patients at risk of depression after ICH, and focus close follow‐up for depression on them. Our study implies that premorbid feelings of depression during the past year is a risk for depression and/or anxiety after stroke, and using simple questions, it would be possible to identify such patients at risk. Obviously, for the diagnosis of depression, further evaluation is needed. The low number of depression diagnoses prior to ICH made this comorbidity less sensitive in identifying patients at risk for post‐ICH depression.

The strength of our study is having extensive data on comorbidities and questioning feelings of depression. The cohort is homogeneous, as the majority of the patients are ethnic Finns, and the utility index values for the HRQoL have country‐specific values. In addition, we used two different methods for the HRQoL evaluation.

The limitations of the study include the rather small cohort and the amount of missing data. The reasons for no consent include death, early transport to another hospital, aphasia and no proxy, and refusal to participate. Dichotomization at the median values has also been used earlier (Delcourt et al., 2017). Due to our small cohort, the utility index values were dichotomized at the median values, in order to get representative sample of patients in both groups. As measuring quality of life is complex, and likely affected by many factors on individual level, it is not surprising, that the models generated were able to explain only 50% of HRQoL. As the past feelings prior to index‐ICH are questioned, it cannot be excluded, that depressive feelings at the time of evaluation affect the results, even though the patients are asked to recall the situation before ICH. The patients were sent the QoL questionnaires at 3 months, and even though the exact time point was not recorded, the analysis represents situation after the acute and subacute phase of the illness. The response rate among patients alive was 56.4%. Taken into consideration the nature of the illness this can be considered quite good, as many of the survivors are not expected to be able to answer due to their neurologic deficits. Also, as expected, the patients not participating in QoL reporting had more severe deficits, and thus our study represents HRQoL of patients with milder deficits. The response rate is in line with survey response rates published in medical journals (Asch et al., 1997).

In conclusion, higher age and parameters that associate with larger strokes, as shown also previously affect the quality of life after ICH. Of comorbidities, chronic heart failure had the strongest association with poor quality of life. Patients reporting feelings of sadness or depression before ICH reported more frequently anxiety and/or depression also after ICH. That had stronger influence on development of depression and/or anxiety than stroke severity‐related and outcome parameters. Our study suggests that a questionnaire on premorbid self‐reported sadness or depression could help to identify patients at risk of depression and/or anxiety after ICH. Obviously, larger prospective studies are needed to confirm our results.

## CONFLICT OF INTERESTS

T.S. has received grants from Orion Corporation (Finland), TEVA Pharmaceutical Industries Ltd., Allergan plc, personal fees from Boehringer Ingelheim Inc., outside the submitted work. D.S. and H.S. report no conflicts of interests.
