# Supplementary material for: Quality of life and depression 3 months after intracerebral hemorrhage
Source: Brain Behav. 2019 Mar 24;9(5):e01270. doi: 10.1002/brb3.1270 (PMC6520301; doi:10.1002/brb3.1270)

Supplemental Table 1. Comparison of the baseline characteristics between patients without (n=96) and with (n=124) data of quality of life (patients known to be alive at 3 months)

| Variable | Excluded (n=96) | Included (n=124) | P Value |
| --- | --- | --- | --- |
| Age, y | 66.0 (53.3-77.8) | 70.5 (62.0-78.0) | 0.050 |
| Male | 61 (63.5) | 65 (52.4) | 0.098 |
| Agreement by proxy | 26 (27.1) | 38 (30.6) | 0.564 |
| mRS 3-5 prior to ICH | 5 (5.2) | 5 (4.0) | 0.751 |
| Diabetes | 16 (16.7) | 16 (12.9) | 0.432 |
| History of ischemic stroke | 6 (6.3) | 15 (12.1) | 0.143 |
| History of any ICH | 5 (5.2) | 7 (5.6) | 0.887 |
| COPD or asthma | 10 (10.4) | 11 (8.9) | 0.699 |
| Dementia | 5 (5.2) | 6 (4.8) | 1.000 |
| Coronary artery disease | 10 (10.4) | 12 (9.7) | 0.856 |
| Atrial fibrillation | 15 (15.6) | 32 (25.8) | 0.068 |
| Chronic heart failure | 4 (4.2) | 8 (6.5) | 0.459 |
| History of cancer | 18 (18.8) | 28 (22.6) | 0.488 |
| Antiplatelet treatment | 21 (21.9) | 26 (21.0) | 0.871 |
| Anticoagulant treatment | 15 (15.6) | 27 (21.8) | 0.250 |
| Antihypertensive medication | 48 (50.0) | 66 (53.2) | 0.635 |
| Antidepressant medication | 4 (4.2) | 4 (3.2) | 0.731 |
| Living alone† | 31 (33.7) | 41 (33.1) | 0.922 |
| Occupational status, working | 29 (30.2) | 27 (21.8) | 0.154 |
| NIHSS on admission | 7.8 (3.0-14.8) | 5.0 (2.3-11.0) | **0.018** |
| GCS on admission | 15.0 (14.0-15.0) | 15.0 (14.0-15.0) | 0.813 |
| Baseline ICH volume, mL | 12.3 (5.8-22.5) | 8.9 (2.6-19.0) | 0.088 |
| ICH location |  |  | 0.307 |
| ICH location, lobar | 25 (26.0) | 36 (29.0) |  |
| ICH location, deep supratentorial | 60 (62.5) | 81 (65.3) |  |
| ICH location, infratentorial | 11 (11.5) | 7 (5.6) |  |
| Deterioration (increase in NIHSS at least 4 points) within 1 week of ICH | 7 (7.3) | 8 (6.5) | 0.806 |
| Acute myocardial infarction or heart failure within 1 week of ICH | 3 (3.1) | 2 (1.6) | 0.655 |
| Any neurosurgery | 5 (5.2) | 5 (4.0) | 0.751 |
| DNR orders in hospital | 18 (18.8) | 17 (13.7) | 0.311 |
| mRS at 3 months |  |  | **0.045** |
| mRS 0 | 4 (4.2) | 5 (4.0) |  |
| mRS 1 | 5 (5.2) | 13 (10.5) |  |
| mRS 2 | 30 (31.3) | 51 (41.1) |  |
| mRS 3 | 18 (18.8) | 22 (17.7) |  |
| mRS 4 | 16 (16.7) | 22 (17.7) |  |
| mRS 5 | 23 (24.0) | 11 (8.9) |  |
| mRS 3-5 at 3 months | 57 (59.4) | 55 (44.4) | **0.030** |
| Barthel Index at 3 months† | 95.0 (35.0-100.0) | 100.0 (85.0-100.0) | **0.013** |
| Living at home at 3 months† | 52 (54.7) | 96 (77.4) | **<0.001** |
| Working at 3 months | 3 (3.1) | 4 (3.2) | 1.000 |
| Living at home at 1 year (vs. sheltered housing)† | 72 (78.3) | 110 (90.9) | **0.010** |
| Working at 1 year† | 8 (8.6) | 13 (10.7) | 0.602 |
| Mortality at 1 year† | 2 (2.1) | 1 (0.8) | 0.582 |

Data are n (%), mean (SE), or median (IQR)

Abbreviations: mRS, modified Rankin Scale; ICH, intracerebral hemorrhage; COPD, chronic obstructive pulmonary disease; NIHSS, the National Institutes of Health Stroke Scale; DNR, do not resuscitate

† Missing data for living alone in 4 patients (1.8 %), Barthel Index in 1 patient (0.5 %), living at home at 3 months in 1 patient (0.5 %), living at home at 1 year in 7 patients (2.7 %), working at 1 year in 6 patients (2.7 %), mortality at 1 year in 5 patients (2.3 %)

Supplemental Figure 1. The distribution of the a) EQ-5D-5L (n=117), and b) 15D (n=108) utility index values at 3 months after ICH.

**A**


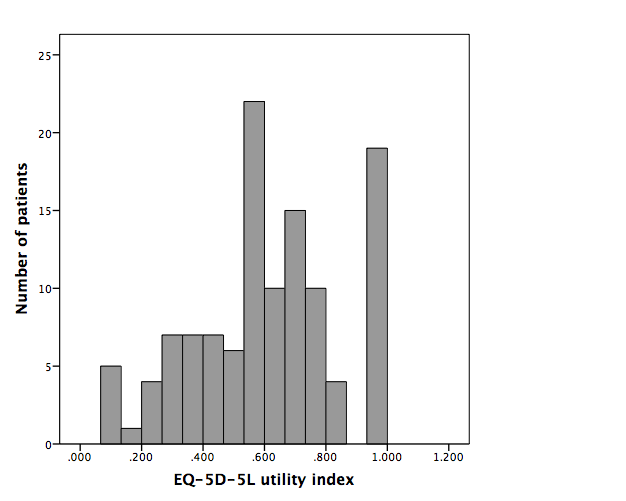


**B**


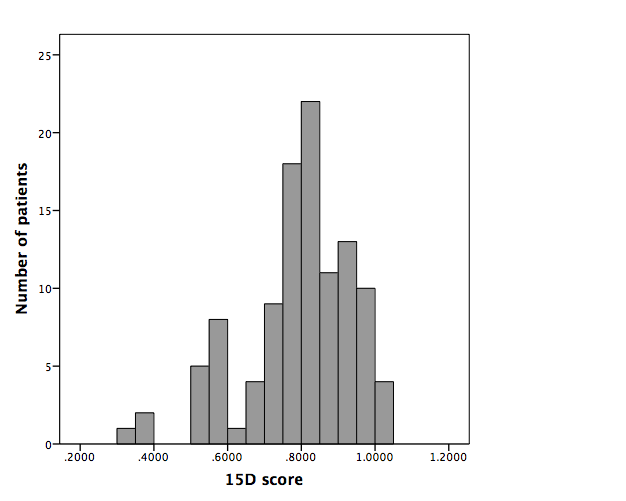


Supplemental Figure 2. a) EQ-5D-5L (n=117), and b) 15D (n=108) utility indexes across mRS groups at 3 months after ICH

**A**


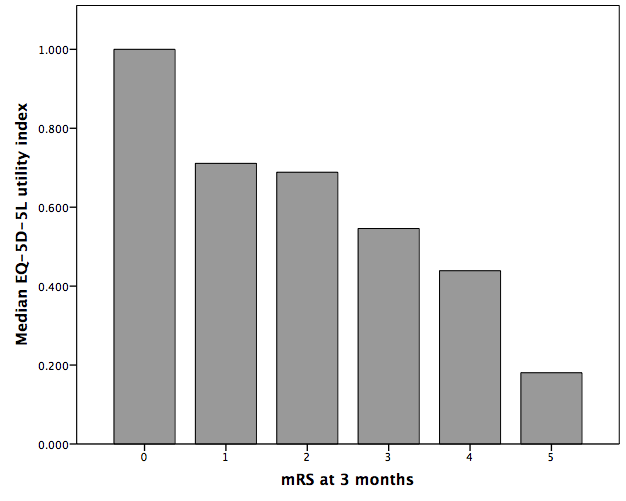


**B**


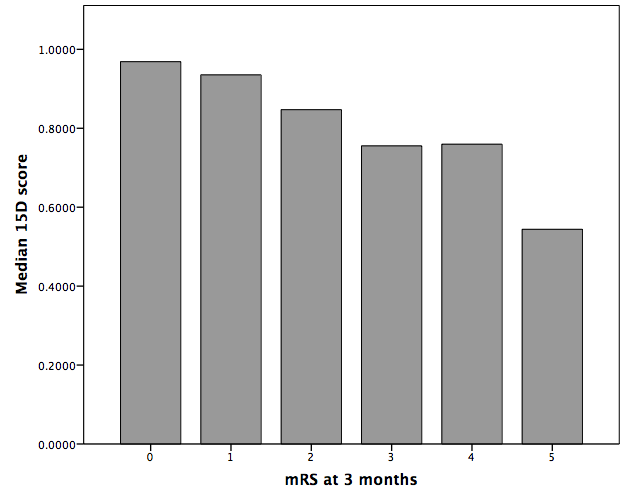

Supplement: Supplementary file 1 [file BRB3-9-e01270-s001.docx]
